# 11-Deoxycorticosterone (DOC)’s Action on the Gill Osmoregulation of Juvenile Rainbow Trout (*Oncorhynchus mykiss*)

**DOI:** 10.3390/biology13020107

**Published:** 2024-02-09

**Authors:** Rodrigo Zuloaga, Luciano Ahumada-Langer, Jorge Eduardo Aedo, Alfredo Molina, Juan Antonio Valdés

**Affiliations:** 1 Departamento de Ciencias Biológicas, Facultad de Ciencias de la Vida, Universidad Andres Bello, Santiago 8370146, Chile; rodrigo.zuloaga.r@gmail.com (R.Z.); lucianofranco.a@gmail.com (L.A.-L.); amolina@unab.cl (A.M.); 2 Interdisciplinary Center for Aquaculture Research (INCAR), Concepción 4030000, Chile; 3 Departamento de Biología y Química, Facultad de Ciencias Básicas, Universidad Católica del Maule, Talca 3466706, Chile; jaedo@ucm.cl

**Keywords:** DOC, mineralocorticoid receptor, stress response, RNA-seq, gills, salmonid

## Abstract

**Simple Summary:**

Aquaculture produces fish that are then marketed to the population, but the type of production used today generates stress in fish. Constant stress, which occurs through the hormone cortisol, negatively affects the seawater adaptation of juvenile fish, which is detrimental to the aquaculture process. For many years, it was thought that only cortisol influenced physiological responses in fish. However, in recent years, the cortisol intermediary 11-deoxycorticosterone (DOC) has been considered a complementary stress-related hormone in fish. Given the above, this work aims to unravel whether DOC is involved in the regulation of early physiological and transcriptional responses in fish gills. To ascertain this, juvenile rainbow trout were injected with DOC and/or pharmacological inhibitors of corticosteroid receptors. Then, the physiological parameters and RNA sequencing of the gills were analyzed. The experiment concluded that DOC is important during stress and that it is vital for the balance between minerals and water in fish. The results of this work will help us understand the other effects of DOC in fish, as well as help improve the monitoring of animal welfare in the aquaculture industry through the incorporation of novel and potential stress molecular biomarkers.

**Abstract:**

In aquaculture, stress can negatively affect fish growth. For years, the cortisol hormone has been thought to play both glucocorticoid and mineralocorticoid functions. Nevertheless, recent research has suggested that 11-deoxycorticosterone (DOC) released during stress could contribute to cortisol actions, though this process is still misunderstood. Here, we evaluated the DOC effects on physiological and early transcriptional responses by RNA-seq. Juvenile rainbow trout were treated with DOC and/or glucocorticoids (mifepristone) or mineralocorticoid (eplerenone) receptor antagonists. Subsequently, plasma was collected, and cDNA libraries were generated from the gills of vehicle (control), DOC, mifepristone, mifepristone with DOC, eplerenone, and eplerenone with DOC groups. Calcium and phosphate levels in plasma were changed. Results revealed 914 differentially expressed transcripts (DETs) induced by DOC compared with control, mainly associated with sodium ion transmembrane transport, gluconeogenesis, negative regulation of transmembrane transport, and activation of innate immune response. DOC versus eplerenone with DOC comparison displayed 444 DETs related to cell-cell junction organization, canonical glycolysis, positive regulation of immune response, and potassium ion transport. Conversely, no DETs were detected in DOC versus mifepristone with DOC comparison. These data suggest that DOC has a relevant role in gill stress response and ion transport, which is differentially regulated by mineralocorticoid receptors.

## 1. Introduction

The aquaculture industry has great potential to solve food problems worldwide [1,2]. This industry currently presents economic losses due to the intensive conditions required for fish farming, which negatively impact fish growth due to several types of stress conditions [3,4]. To overcome a stressful event, teleost fish possess physiological mechanisms to respond through a neuroendocrine adaptative reaction [5]. The neuroendocrine stress response in fish begins with activating the hypothalamic–pituitary–interrenal axis (HPI), which secretes glucocorticoid hormones into the bloodstream to regulate the physiological and metabolic responses that occur in order to maintain homeostasis [6].

Cortisol, which is the ligand of two glucocorticoid receptors (GR1 and GR2), is the main glucocorticoid hormone synthesized by fish. In addition, it is a mineralocorticoid receptor (MR) [7]. The MR in mammals serves as the main aldosterone receptor; however, though this hormone cannot be synthesized by fish, its receptor can [8]. Cortisol has been thought to function as a glucocorticoid and mineralocorticoid in fish for many years [9,10,11]. However, recent results have suggested that 11-deoxycorticosterone (DOC), as an MR ligand, may exert physiological effects as a mineralocorticoid [8,12,13]. DOC is a corticosteroid hormone that is found circulating in the bloodstream of fish in small amounts, and it also modulates the activity of MRs [14]. Like cortisol, DOC synthesis comes from progesterone via the enzymatic activity of 21β-hydroxylase, and it is subsequently secreted by the interrenal cells of the anterior kidney [15,16]. In rainbow trout (*Oncorhynchus mykiss*), DOC has been found to participate in the endocrine regulation of spermiation/spermatogenesis that occurs in teleost fish [14,16]. More recently, a regulation of confinement stress that suggests a role during the stress response was also found in trout [17]. In this sense, it has been described that one of the main processes affected during stress is osmoregulation, which is key in the smoltification of juvenile salmonids and one of the most critical phases in salmon aquaculture [18]. Nevertheless, the role of DOC in osmoregulation is poorly understood.

Osmoregulation is the process responsible for maintaining homeostasis between the concentration of solutes and water flow from the inside to the outside [19,20]. In this context, the gills are the main osmoregulatory organ of fish, which have two ways of regulating hydromineral flow: (i) transcellular transport, which occurs through transmembrane pumps (such as sodium–potassium (Na^+^/K^+^ATPase) [21]), ionic cotransporters (such as Na^+^-K^+^-2Cl^−^ (NKCC1) [22]), and water cotransporters (such as aquaporins [19,20]); and (ii) paracellular transport where the movement of solutes occurs between epithelial cells along the intercellular space, which occurs via diffusion through tight junction proteins (claudin/occludin) [23,24]. Until the present, it has been unclear how osmoregulation in fish would be regulated during a DOC-induced stress response. Moreover, the participation of DOC in other relevant gill responses remains unknown. Certain studies have shown that the effects of DOC on this process are minimal compared with cortisol [25,26]. However, other research in salmonids has indicated that though DOC does not affect Na^+^/K^+^ATPase activity [25], it can increase the α1 isoform transcriptional levels of this pump in gill explants [26]. According to this, we hypothesize that DOC can regulate the expression of osmoregulation-related genes via MR. The present work evaluates the effects of DOC on physiological and early transcriptional responses in juvenile rainbow trout. For this, we performed a transcriptomic and physiological analysis to evaluate the global response of the fish gills treated with DOC in the presence or absence of specific GR and MR antagonists. The data revealed changes in ion plasma levels and biological processes related to DOC-modulated osmoregulation via MR.

## 2. Materials and Methods

### 2.1. Experimental Protocol

This study followed animal welfare protocols, and it was authorized by the bioethical committees (protocol code 012/2020) of the Universidad Andres Bello and the National Commission for Scientific and Technological Research of the Chilean government. More information regarding how the assays were conducted is detailed in Zuloaga et al. [27]. Thirty juvenile rainbow trout (with an average weight of 15.4 g ± 0.8) were kept at a natural temperature of 14 °C ± 1 °C in light conditions of a 12 h light cycle and a 12 h dark cycle, and they were fed with commercial pellets. The fish were sedated with benzocaine (25 mg/L, #BZ^®^-20, Veterquimica, Maipú, RM, Chile) and injected with metyrapone (#M2696, Sigma-Aldrich, St. Louis, MO, USA) at a dose of 1 mg per kilogram in the abdominal cavity for one hour. Then, the fish were divided into six groups (*n* = 5 per group). The first group received DMSO-PBS 1X (vehicle) and the second group received 11-deoxycorticosterone acetate (#56-47-3, DOC, USBiological, Salem, MA, USA) at physiological concentrations of 1 mg per kilogram. The third and fourth groups were treated with mifepristone (RU486, #M8046, Sigma-Aldrich) at a dose of 1 mg per kilogram, and the fourth group also received DOC at a dose of 1 mg per kilogram. Last, the fifth and sixth groups were treated with eplerenone (#107724-20-9, Santa Cruz Biotech., Santa Cruz, CA, USA) at a dose of 1 mg per kilogram, and the sixth group also received DOC at a dose of 1 mg per kilogram. Three hours after the treatments, the rainbow trout were euthanized using benzocaine at a concentration of 300 mg/L. Blood samples were collected from the caudal vessel using a 1 mL syringe with heparin (#9041-08-1, Santa Cruz Biotech.) at a concentration of 10 mg/mL. Plasma was obtained by centrifuging the blood at 5000× *g* for 10 min. The plasma and gills sampled were rapidly frozen using liquid nitrogen and then stored at −80 °C.

### 2.2. Measurement of the Solutes in Plasma

The contents of phosphate (PO_4_^−^, #BML-AK111, BIOMOL^®^ Green, Enzo, Farmingdale, NY, USA), calcium (Ca^+2^, #MAK022, Sigma-Aldrich), and chloride (Cl^−^, #MAK023, Sigma-Aldrich) from the plasma were quantified using colorimetric assays following the manufacturer’s instructions. For the phosphate, the linear range of detection was between 0.03–2 nmol/well. For the calcium, the linear range of detection was between 0.4–2.0 μg/well. For the chloride, the linear range of detection was between 20–100 nmol/well. All the analytes were first evaluated and validated in the plasma from other teleost fish [28,29,30].

### 2.3. Measurement of the Muscle Water Content

The muscle moisture was measured to evaluate the physiological response of the fish in response to DOC-induced stress. For this, 0.1 g of muscle tissue was weighed, which was then incubated at 56 °C overnight. The next day, the tissue was weighed again. The water content was obtained by calculating the difference between the wet and dry weight of the muscle tissue [31].

### 2.4. Library Construction and Sequencing

RNA was isolated (using an EZNA^®^ Total RNA Kit (#R6834-00S, OMEGA Bio-Tek, Norcross, GA, USA) in accordance with the manufacturer’s instructions) from the gills (0.1 g) of the following groups: vehicle, DOC, mifepristone, mifepristone + DOC, eplerenone, and eplerenone + DOC. Following this, the RNA Clean & Concentrator™-5 (with DNase I) kit (#R1013, Zymo Research, Orange, CA, USA) was used. The quality of the RNA was assessed using a capillary electrophoresis Fragment Analyzer Automated CE System (Advanced Analytical Technologies, Ames, IA, USA). RNA samples with RQN values greater than or equal to 8 were chosen for library construction. The total RNA quantity was determined using a fluorometer and a Qubit RNA BR assay kit (#Q10210, Invitrogen, Carlsbad, CA, USA). For each condition, 1 µg of RNA was used to generate twelve cDNA libraries with a TruSeq RNA Sample Preparation kit v2 (#RS-122-2001, Illumina, San Diego, CA, USA), which was then quantified with a Kapa Library Quantification kit (#kk4824, Roche, NJ, USA) on an AriaMx real-time PCR (qPCR) thermocycler (Agilent, Santa Clara, CA, USA). In addition, the library size was determined by capillary electrophoresis. The resulting twelve libraries were sequenced on a Hiseq X (Illumina) platform at Macrogen (Seoul, Korea) using a paired-end strategy (2 × 150 bp).

### 2.5. Raw Data Processing, RNA-Seq Analysis, and Functional Annotation Analysis

The quality control of the raw reads was measured with a FastQC v0.11.9 (https://www.bioinformatics.babraham.ac.uk/projects/fastqc/, accessed on 5 August 2023). Fastp software [32] was used to remove adapters and discard low-quality reads (Q < 30), low complexity filters (y), and poly X trimming in the 3′ ends. Quality reads were de-duplicated with cd-hit-dup software v4.8.1 [33]. Then, the high-quality reads were separately mapped onto a rainbow trout reference genome OmykA_1.1 (GCF_013265735.2) with HISAT2 v2.2.1 [34], which was composed of a 71,413-coding sequence (CDS) with default mapping parameters. Finally, the mapped reads were sorted and transformed into BAM format using Samtools v1.6 [35].

The in silico differential expression analysis was based on reads uniquely mapped to the reference and proportional-based statistical K-tests. The counting was performed with FeatureCounts from the R package Rsubread v2.8.1 [36]. The raw transcript count matrix was filtered to remove the low-quantity transcripts (counts > 10). Then, R package DESeq2 v1.34 [37] was used to determine the differentially expressed transcripts (DETs) (padj < 0.05 and absolute Log2FC > 1). A comparison between the vehicle and DOC groups considered the potential DETs regulated by DOC. Comparisons between the DOC and mifepristone plus DOC groups, as well as the DOC and eplerenone plus DOC groups, were achieved by considering the potential DETs regulated by DOC and mediated by the glucocorticoid and mineralocorticoid receptors, respectively. As the reference genome was not fully annotated, a custom annotation of the mapped CDS was created with the eggNOG-mapper using the eggNOG 5 database [38]. The IDs of DETs were extracted along with custom annotation and used as the input for the topGO v2.46 enrichment analysis [39]. The total genes were used to provide a background for statistical analysis. Subsequently, a topGO object was generated in R software v.4.1.2, and the ontological enrichment analysis of the biological processes, molecular functions, and cellular components of the up and down genes regulated by each of the group comparisons was carried out using Fisher’s test. Finally, the data representation was conducted using the R package ggplot2 v3.4.3 [40].

### 2.6. Real-Time PCR Validation

Rainbow trout gills were used to extract, concentrate, and purify the total RNA samples, as mentioned previously. The RNA samples were then quantified using Nanodrop technology (BioTek, Winooski, VT, USA), and their quality was assessed through agarose gel electrophoresis with a 1.2% formaldehyde solution. Total RNA (1 μg) was converted into cDNA using the ImProm-II™ Reverse Transcription System (#A3800, Promega, Madison, WI, USA). Primers for amplifying the candidate genes were designed using PrimerQuest software (https://www.idtdna.com/pages/tools/primerquest, accessed on 25 October 2023) and validated using Beacon Designer™ Free Edition (http://www.premierbiosoft.com/qpcr/index.html, accessed on 25 October 2023). The qPCR was conducted using a reaction mixture that contained 7.5 μL of 2× Brilliant II SYBR^®^ master mix (#600828, Agilent), 6 µL of cDNA (20-fold diluted), and 0.75 μL of each primer (250 nM) in a final volume of 15 µL. Control reactions included a no-template control (NTC) and a control without reverse transcriptase (noRT). Supplementary Table S1 provides a list of the primers used in this study. The amplification process was carried out in triplicate with the following thermal cycling conditions: initial activation at 95 °C for 5 min, followed by 40 cycles of denaturation at 95 °C for 15 s, annealing at 58–67 °C for 15 s, and elongation at 72 °C for 15 s. A melting curve analysis was included to confirm the presence of a single PCR product. The 2^−ΔΔCT^ method was used for relative gene quantification [41], and the results were expressed as a fold change compared with the vehicle or DOC group. Beta-actin (*actβ*) and 40S ribosomal protein S30 (*fau*) were used as housekeeping genes.

### 2.7. Statistical Analysis

The data were analyzed utilizing a normal (Gaussian) distribution test and a Kolmogorov–Smirnov normality test. Then, data variance was analyzed by one-way ANOVA. This was followed by Tukey’s honest significant difference as a post-test, which was conducted employing Graph Prism 8.0 software (San Diego, CA, USA). A significance level of *p* < 0.05 was employed to determine statistical significance.

## 3. Results

### 3.1. Evaluation of Solutes in the Plasma, Muscle Water Content, and Glycogen Content in the Gills

**Figure 1:**
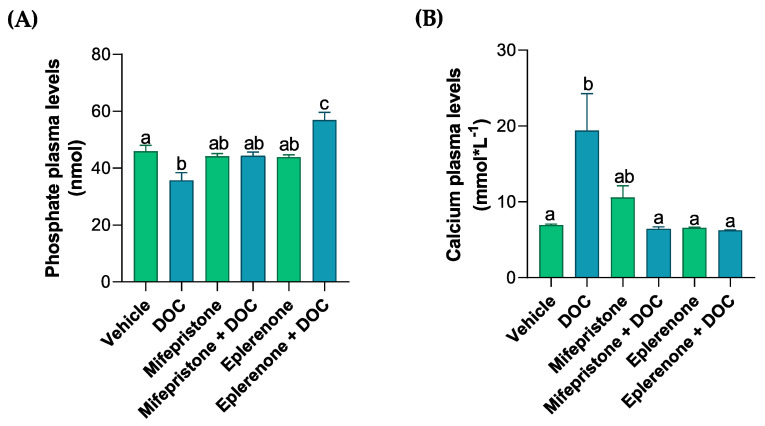

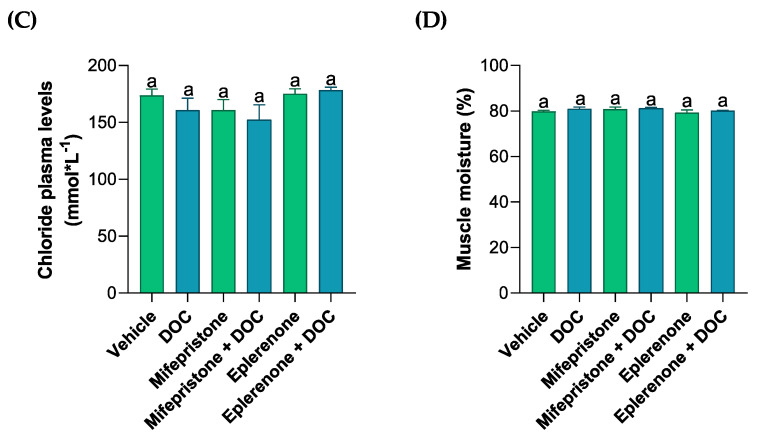
The plasma phosphate, calcium, chloride, and muscle water content of the rainbow trout. The phosphate (**A**), calcium (**B**), and chloride (**C**) plasma levels, as well as the (**D**) muscle water content, were measured in the fish treated with the vehicle, DOC, mifepristone, mifepristone + DOC, eplerenone, and eplerenone + DOC. The results are expressed as the means ± standard error of the means (SEM) (*n* = 5). Different letters represent the significant differences (*p* < 0.05) present when groups were compared.

The plasma levels of different ions (PO_4_^−^, Ca^+2^, Cl^−^, Figure 1A–C) were measured by colorimetric assays in order to analyze the solutes related to the osmoregulation induced by DOC in rainbow trout. Phosphate plasma levels decreased with DOC treatment (35.71 nmol ± 2.75, Figure 1A) compared with the vehicle group. These effects were reversed and increased significantly when treated with DOC plus the MR inhibitor (56.92 nmol ± 2.73) when compared with the DOC group, and this was even more pronounced when compared with the vehicle group. The calcium levels induced by DOC (19.41 nmol·L^−1^ ± 4.86, Figure 1B) increased significantly versus the vehicle group, and this effect was reversed when mifepristone plus DOC (6.4 nmol·L^−1^ ± 0.3) and eplerenone plus DOC (6.23 nmol·L^−1^ ± 0.07) were added, in comparison with the DOC group. However, the plasma chloride levels did not change compared with the vehicle or DOC group (Figure 1C). Additionally, the muscle water content was measured to evaluate the physiological response induced by the DOC in rainbow trout (Figure 1D). The results showed that there were no significant changes in all the groups when compared with the vehicle or DOC groups (Figure 1D).

### 3.2. Transcriptomic Responses of the Rainbow Trout Gill to DOC Mediated by the Mineralocorticoid Receptor (MR)

The transcriptomic response induced by DOC when modulated by the GR and MR was evaluated through RNA sequencing of the gills of rainbow trout that were treated for 3 h with the vehicle, DOC, mifepristone, mifepristone + DOC, eplerenone, and eplerenone + DOC. A total of 910,741,404 reads were obtained, and they corresponded with 12 cDNA libraries. After trimming, 844,057,348 high-quality reads were obtained and used for additional RNA-seq analysis. A total of 731,291,286 reads (86.64%) were mapped against the 71,413 CDS of the rainbow trout. Details on the low-quality read removal and mapping are shown in Supplementary Table S2.

**Figure 2:**
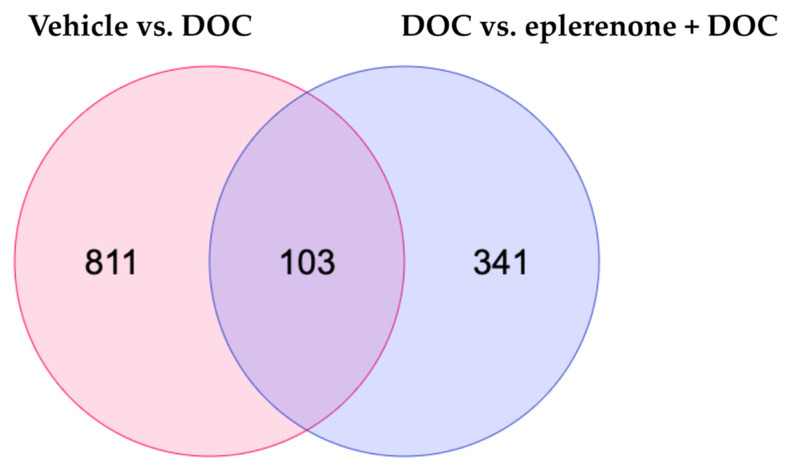
Venn diagram analysis. The Venn diagram indicates the numbers of differentially expressed transcripts under the vehicle vs. DOC and the DOC vs. eplerenone + DOC treatments (padj < 0.05 and absolute Log2FC > 1).

To detect the DETs, the samples were exposed to paired comparisons against the vehicle vs. DOC group, DOC vs. mifepristone + DOC, and DOC vs. eplerenone + DOC. For the first group, 914 DETs were found, with 455 up and 459 down. For the DOC vs. mifepristone + DOC, there were only 2 DETs detected; as such, we excluded this group from the analysis. Finally, for the DOC vs. eplerenone + DOC comparison, 444 DETs were found, with 313 up and 131 down. The complete list of DETs in the vehicle vs. DOC, DOC vs. mifepristone + DOC, and DOC vs. eplerenone + DOC comparisons is detailed in Supplementary Table S3. A Venn diagram analysis showed that there were 103 DETs in common between the comparisons, 811 DETs were unique for the vehicle vs. DOC comparison, and 341 DETs were unique for the DOC vs. eplerenone + DOC comparison (Figure 2).

**Figure 3:**
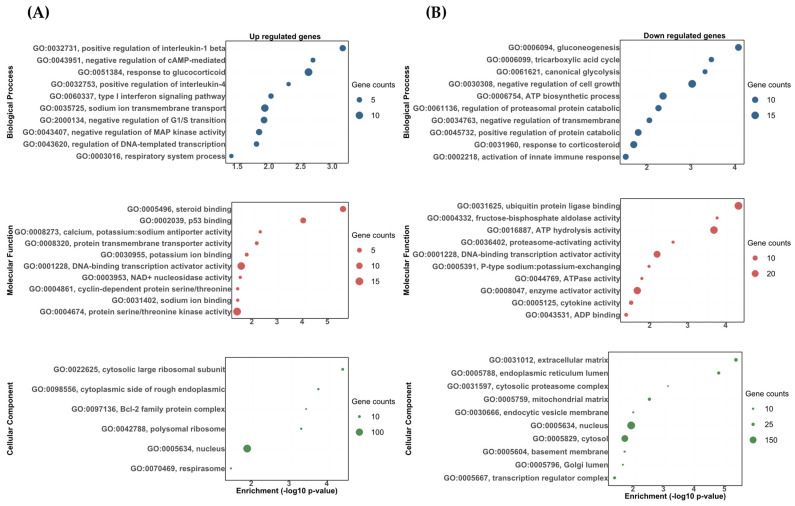
The gene ontology biological process (BP), molecular function (MF), and cellular component (CC) enrichment of the DETs in the vehicle and DOC treatments. (**A**) Upregulated DETs. (**B**) Downregulated DETs. The graph indicates the −log_10_(*p*-value) that was enriched of the differentially expressed transcripts between the vehicle and DOC groups with a padj of <0.05.

**Figure 4:**
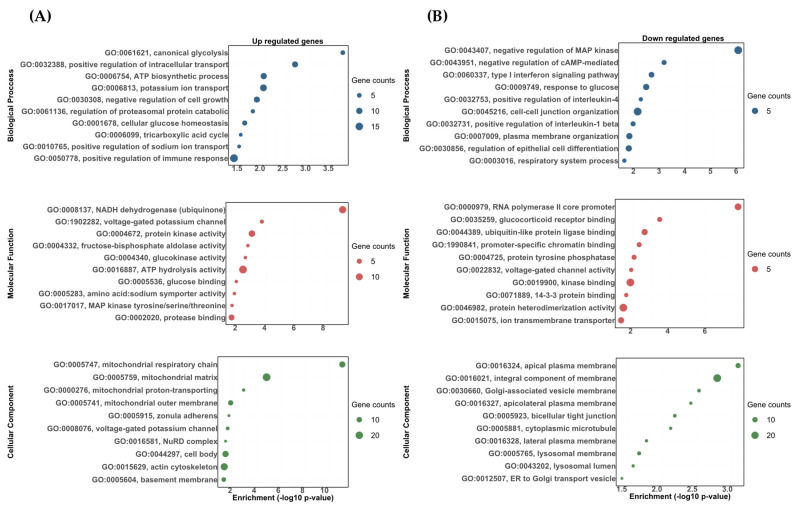
The gene ontology biological process (BP), molecular function (MF), and cellular component (CC) enrichment of the differentially expressed transcripts between the DOC vs. eplerenone + DOC treatments. (**A**) Upregulated DETs. (**B**) Downregulated DETs. The graph indicates the −log_10_(*p*-value) that was enriched by the differentially expressed transcripts between the group DOC vs. eplerenone + DOC with a padj of <0.05.

The DETs were analyzed using topGO software and classified as either biological processes, molecular functions, or cellular components. The DETs were then significantly enriched in a variety of biological processes, molecular functions, and cellular components. Most of these categories showed the upregulated and downregulated transcripts correlated to the stress response, carbohydrate metabolism, osmoregulation, and immune response (Figure 3 and Figure 4). Among the upregulated transcripts in the vehicle vs. DOC comparison, we found biological processes related to responses to glucocorticoids (GO:0051384), sodium ion transmembrane transport (GO:0035725), the positive regulation of interleukin-1 beta production (GO:0032731), the type I interferon signaling pathway (GO:0060337), and the regulation of DNA-templated transcriptions in response to stress (GO:0043620) (Figure 3A). Conversely, in the downregulated ones, we found biological processes related to the ATP biosynthetic process (GO:0006754) in response to corticosteroids (GO:0031960), gluconeogenesis (GO:0006094), the positive regulation of the protein catabolic process (GO:0045732), the negative regulation of transmembrane transports (GO:0034763), and canonical glycolysis (GO:0061621) (Figure 3B). For the molecular functions, the most impacted GO terms were assigned to potassium ion binding (GO:0030955), calcium, potassium:sodium antiporter activity (GO:0008273), and sodium ion binding for upregulated transcripts (GO:0031402) (Figure 3A). Furthermore, cytokine activity (GO:0005125), fructose-bisphosphate aldolase activity (GO:0004332), and P-type sodium:potassium-exchanging transporter activity (GO:0005391) were found for the downregulated transcripts (Figure 3B). Finally, for the cellular components, the most impacted GO terms were assigned to a nucleus (GO:0005634), cytosolic large ribosomal subunits (GO:0022625), and the cytoplasmic side of rough endoplasmic reticulum membranes (GO:0098556) for upregulated transcripts (Figure 3A). In addition, a nucleus (GO:0005634), cytosol (GO:0005829), and an extracellular matrix (GO:0031012) were found for the downregulated transcripts (Figure 3B).

For the DOC vs. eplerenone + DOC comparison, the following biological processes were found to be upregulated: the positive regulation of the immune response (GO:0050778), the potassium ion transport (GO:0006813), the ATP biosynthetic process (GO:0006754), and the positive regulation of sodium ion transports (GO:0010765) (Figure 4A). In the downregulated ones, we found biological processes related to cell–cell junction organization (GO:0045216), the response to glucose (GO:0009749), the type I interferon signaling pathway (GO:0060337), and the positive regulation of interleukin-1 beta production (GO:0032731) (Figure 4B). For the molecular functions, the most impacted GO terms in the upregulated transcripts were assigned to ATP hydrolysis activity (GO:0016887), protein kinase activity (GO:0004672), the voltage-gated potassium channel activity involved in ventricular cardiac muscle cell action potential repolarization (GO:1902282), and the glucose binding for (GO:0005536) (Figure 4A). On the other hand, the most impacted GO terms in the downregulated transcripts were assigned to kinase binding (GO:0019900), ion transmembrane transporter activity (GO:0015075), glucocorticoid receptor binding (GO:0035259), and voltage-gated channel activity (GO:0022832) (Figure 4B). Finally, for the cellular components, the most impacted GO terms were assigned to the mitochondrial matrix (GO:0005759), actin cytoskeleton (GO:0015629), voltage-gated potassium channel complex (GO:0008076), and zonula adherens (GO:0005915) for the upregulated transcripts (Figure 4A). Furthermore, the integral component of the membranes (GO:0016021), lysosomal membranes (GO:0005765), bicellular tight junctions (GO:0005923), and cytoplasmic microtubules (GO:0005881) were found for the downregulated transcripts (Figure 4B). All the enriched upregulated and downregulated biological processes, molecular functions, and cellular components between the vehicle vs. DOC and DOC vs. eplerenone + DOC comparisons are shown in Supplementary Table S4 and Supplementary Table S5, respectively.

### 3.3. Validation of the In Silico Data by Real-Time PCR

**Figure 5:**
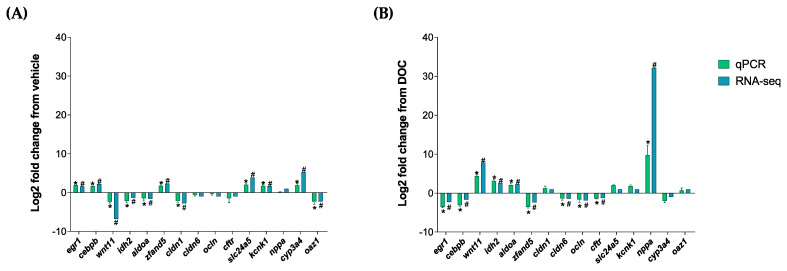
RNA-seq analysis validation via a real-time PCR of the DETs. The following DETs were selected for the real time-PCR validation of the RNA-seq process: *egr1*, *cebpb*, *wnt11*, *idh2*, *aldoa*, *zfand5*, *cldn1*, *cldn6*, *ocln*, *cftr*, *slc24a5*, *kcnk1*, *nppa*, *cyp3a4*, and *oaz1*. (**A**) Validation results between the vehicle vs. DOC and (**B**) the DOC vs. eplerenone + DOC. For the RNA-seq, “#” in blue indicates a padj of <0.05 and an absolute Log2FC of >1. For the real-time PCR (*n* = 3), the relative expression was normalized against *actβ* and *fau*, and the “*” in green indicates the significant differences in the fold change in the vehicle or DOC groups (mean ± SEM, *n* = 3, *p* < 0.05). The genes had the following abbreviations: *egr1* (early growth response 1); *cebpb* (CCAAT enhancer binding protein beta); *wnt11* (Wnt family member 11); *idh2* (isocitrate dehydrogenase (NADP(+)) 2); *aldoa* (aldolase, fructose-bisphosphate A); *zfand5* (zinc finger AN1-type containing 5); *cldn1* (claudin 1); *cldn6* (claudin 6); *ocln* (occludin); *cftr* (cystic fibrosis transmembrane conductance regulator); *slc24a5* (solute carrier family 24 member 5); *kcnk1* (potassium two-pore domain channel subfamily K member 1); *nppa* (natriuretic peptide A); *cyp3a4* (cytochrome P450 family 3 subfamily A member 4); *oaz1* (ornithine decarboxylase antizyme 1); *fau* (40S ribosomal protein S30); and *actβ* (beta-actin).

The validation of the RNA-seq analysis, as well as of the participation of the GR and MR in the different enriched biological processes, was carried out with the representative DETs of the following groups: vehicle vs. DOC and DOC vs. eplerenone + DOC. We selected the following 15 DETs for both of the comparisons: early growth response 1 (*egr1*); CCAAT enhancer binding protein beta (*cebpb*); Wnt family member 11 (*wnt11*); isocitrate dehydrogenase (NADP(+)) 2 (*idh2*); aldolase, fructose-bisphosphate A (*aldoa*); zinc finger AN1-type containing 5 (*zfand5*); claudin 1 (*cldn1*); claudin 6 (*cldn6*); occludin (*ocln*); cystic fibrosis transmembrane conductance regulator (*cftr*); solute carrier family 24 member 5 (*slc24a5*); potassium two-pore domain channel subfamily K member 1 (*kcnk1*); natriuretic peptide A (*nppa*); cytochrome P450 family 3 subfamily A member 4 (*cyp3a4*); and ornithine decarboxylase antizyme 1 (*oaz1*). These DETs were then validated by real-time PCR (Figure 5). Figure 5A shows the comparison results of the real-time PCR validation of the vehicle vs. DOC group with a high Pearson correlation of r = 0.8845 (*p*-value < 0.001). The PCR validation results of the comparison between the DOC vs. eplerenone + DOC groups, which also presented an elevated Pearson correlation of r = 0.8726 and a *p*-value of <0.001, are shown in Figure 5B.

## 4. Discussion

In this work, we performed a physiological and transcriptomic analysis of juvenile rainbow trout that were treated with DOC and/or specific GR and MR antagonists. The data revealed that DOC-induced physiological effects on the ion plasma levels (calcium and phosphate) and DETs related to ion transmembrane transport demonstrated osmoregulation not only in gills but also in carbohydrate metabolism (glycolysis/gluconeogenesis) and innate immune response via MR. As mentioned before, unlike mammals, fish do not synthesize aldosterone, even when they express MRs in different tissues [42,43,44]. In this context, it has been proposed that cortisol functions as a glucocorticoid and mineralocorticoid in fish homeostasis; however, recent research has suggested that DOC through MRs may act as mineralocorticoids during stress [17,27]. As we expected, an enriched biological process related to the regulation of DNA-templated transcription in response to stress (*cebpb* and *erg1*) supported a stress response that was induced by the DOC in the gills. No less important is the differential expression related to the response to corticosteroids (*cldn1*) and glucocorticoids (*cyp3a4*), which could also indicate that DOC may directly modulate their expression through MRs.

Regarding the regulation of ion plasma levels that are mediated by DOC, we found that this hormone promotes an increase in calcium plasma levels in rainbow trout. Interestingly, other glucocorticoids, such as cortisol, induced a rapid calcium release in several fish, including rainbow trout [45,46]. Moreover, we observed that pretreatment with the MR antagonist abolished the DOC-induced calcium increase in the plasma. In this context, early MR activation was associated with rapid changes in the ion transport in the tubular epithelial cells of the kidney in mammals, as well as changes in intracellular calcium levels [47]. Even though the role of DOC is still controversial, we suggest that DOC via MRs is involved in the rapid calcium flux in rainbow trout. Conversely, the administration of DOC decreased the phosphate plasma levels, which were reverted with the MR inhibitor. To the best of our knowledge, there have not been any studies that have evaluated these ions in fish plasma under DOC treatment. Nevertheless, there have been previous reports that have described cortisol as inducing variations in the bone mineral metabolism of fish during a stress response through GR, which has subsequently affected calcium/phosphorus homeostasis [48,49,50]. Therefore, we speculate that DOC, as well as cortisol, contributes to these mechanisms by modulating the ion flux in gills. Conversely, DOC does not affect plasma chloride levels or muscle water content, thus suggesting that this intermediary does not present the similar effects that were previously determined by cortisol [51,52]. We found a chloride cotransporter that was differentially expressed in gills (*cftr*), which could suggest that certain transcriptional changes are not reflected at the physiological level. Therefore, further experiments are required to determine these effects.

Several in vivo and in vitro studies have demonstrated the role of cortisol in osmoregulation processes that provide salinity tolerance as well as participate in both ion uptake and salt secretion in teleost fish [53,54,55]. However, the role of DOC in these processes is less understood. We found that the DOC obtained via MR is involved in the regulation of several osmoregulatory-related genes in rainbow trout gills, including several ion cotransporters (*cftr*, *slc24a5*, and *kcnk1*) that are involved in transcellular ion transport, as well as tight junction proteins (*cldn1*, *cldn6*, and *ocln*) that are related to paracellular ion transport. These suggest that DOC could participate in both processes. In line with these results, it was found that DOC treatment increases both the *α-1a* and *α-1b* subunit isoforms of Na^+^/K^+^ATPase on the gill explants of Atlantic salmon [26]. However, DOC does not participate in the regulation of other relevant ion cotransporters, such as NKCC and CFTR on the gill explants of rainbow trout [56]. In relation to this, a review of the mineralocorticoid signaling role in teleost fish argued that this minor action of DOC on osmoregulation exists, but it did not find information on its effects on tight junction proteins [57]. To the best of our knowledge, the present study could provide the first instances of evidence that DOC-MR also has a role in paracellular ion transport. Taken together, we propose that DOC could be a potential regulator of several osmoregulatory mechanisms in fish.

To obtain a complete landscape of the gill transcriptional response mediated by DOC, we performed an integrative RNA-seq analysis. We found that relevant biological processes, such as immunity and metabolism, are potentially regulated by DOC. Regarding the immune response, the identified DETs were found to participate in pro-inflammatory cytokine production (*nppa* and *egr1*) [58,59]. In agreement with these findings, Mathieu et al. determined that DOC increases the expression of several immune-related genes, such as *C-type lysozyme* and *apolipoprotein A1* in fish gills [60,61]. By considering this, together with our RNA-seq analysis, we can support that DOC has a role as a potential immune stimulator in fish. Regarding metabolism, we found that biological processes related to the ATP biosynthesis of glycolysis/gluconeogenesis (*aldoa* and *idh2*) could indicate a need for an energy supply for stress conditions in fish [62,63]. In agreement with these findings, Milla et al. found that DOC induces physiological and proteomic responses related to the immune response in the spleen of Eurasian perch (*Perca fluviatilis*), as well as in the proteins involved in the Krebs cycle and glucose metabolism in the liver [64]. Carbohydrate metabolism appears to play an important part in the energy supply for osmoregulation [65], which is crucial during stress. In this regard, it was recently found that there is a metabolite translocation between tilapia gill ionocytes and neighboring glycogen-rich (GR) cells [66], thereby indicating a significant participation in the local energy supply of gills for osmoregulatory mechanisms. Hence, we suggest that DOC could also possess a function in the carbohydrate metabolism of fish gills.

Finally, we recently determined that DOC is also capable of inducing an early transcriptional response in the skeletal muscle of rainbow trout [27]. These effects are differentially modulated by GR and MR, and they present enriched biological processes that are related to cell differentiation and autophagy, respectively. Nevertheless, this tissue presents a limited number of processes that are regulated by DOC; meanwhile, we showed more diverse effects with specific actions via MRs on gills. The data could indicate a tissue-specific effect and further complex physiological responses during short-term stress. These observations are in line with previous reports on teleost fish, where GRs and MRs presented contrasting actions in the target tissues [66,67,68]. Faught and Vijayian observed that MRs promote anabolic processes in glucose metabolism as well as curtail the catabolic effects of GR, during stress in zebrafish muscle via cortisol [67]. With respect to gills, a recent study revealed a specific impact of MR, and not GR, on the salinity acclimation and ionocyte development in tilapia [68]. Thus, this information could support the hypothesis of a complementary action of DOC to cortisol during a stress response. Nevertheless, considering that plasmatic DOC levels are present during different life stages of fishes, the potential effects of this hormone on the stress response of adult fish is an interesting research topic for future analyses.

## 5. Conclusions

There are limited studies that have evaluated DOC’s effects on gills where the focus has been on the regulation of specific water and ion transporters during changes in salinity environments. This study investigated the broad effects of novel stress-related glucocorticoids in this tissue, thereby revealing the potential DOC-modulated target genes and biological events involved not only in osmoregulation but also in carbohydrate metabolism- and innate immune-related processes. We propose that DOC, potentially through MR signaling, is an important regulator of physiological and transcriptional responses in rainbow trout gills. This work may contribute to the understanding of the other physiological effects of DOC in fish, and it could help the aquaculture industry to improve the monitoring of animal welfare through the incorporation of novel and potential stress molecular biomarkers.

## Data Availability

The raw read sequences obtained from sequencing were deposited in the Sequence Read Archive (SRA) under BioProject accession number PRJNA1046010 (SRR26967251, SRR26967250, SRR26967246, SRR26967249, SRR26967248 and SRR26967247). The datasets generated and analyzed during the current study are not publicly available due to privacy or ethical restrictions but are available from the corresponding author upon reasonable request.

## Supplementary Materials

The following supporting information can be downloaded at: https://www.mdpi.com/article/10.3390/biology13020107/s1; Table S1: List of primer sequences used in real-time time-PCR for RNA-seq validation; Table S2: Summary of RNA sequencing and mapping for rainbow trout gills; Table S3: Complete list of DETs in vehicle vs. DOC, DOC vs. mifepristone + DOC and DOC vs. eplerenone + DOC comparisons; Table S4: Enrichment of DETs associated to biological process, molecular functional, and cellular components in vehicle vs. DOC comparison. Table S5: Enrichment of DETs associated with biological process, molecular functional, and cellular components in DOC vs. eplerenone + DOC comparison.
